# Up-regulation of stomatin expression by hypoxia and glucocorticoid stabilizes membrane-associated actin in alveolar epithelial cells

**DOI:** 10.1111/jcmm.12069

**Published:** 2013-05-15

**Authors:** Ji-Cheng Chen, Hao-Yu Cai, Yan Wang, Yuan-Yuan Ma, Liang-Nian Song, Li-Juan Yin, Dong-Mei Cao, Fei Diao, Yi-Dong Li, Jian Lu

**Affiliations:** a Department of Pathophysiology, The Second Military Medical UniversityShanghai, China; b Department of Medicine, Herbert Irving Comprehensive Cancer Center, Columbia University Medical CenterNew York, NY, USA

**Keywords:** actin cytoskeleton, alveolar epithelial cells, dexamethasone, hypoxia, stomatin

## Abstract

Stomatin is an important lipid raft-associated protein which interacts with membrane proteins and plays a role in the membrane organization. However, it is unknown whether it is involved in the response to hypoxia and glucocorticoid (GC) in alveolar epithelial cells (AEC). In this study we found that hypoxia and dexamethasone (dex), a synthetic GC not only up-regulated the expression of stomatin alone, but also imposed additive effect on the expression of stomatin in A549 cells, primary AEC and lung of rats. Then we investigated whether hypoxia and dex transcriptionally up-regulated the expression of stomatin by reporter gene assay, and found that dex, but not hypoxia could increase the activity of a stomatin promoter-driven reporter gene. Further deletion and mutational studies demonstrated that a GC response element (GRE) within the promoter region mainly contributed to the induction of stomatin by dex. Moreover, we found that hypoxia exposure did not affect membrane-associated actin, but decreased actin in cytoplasm in A549 cells. Inhibiting stomatin expression by stomatin siRNA significantly decreased dense of peripheral actin ring in hypoxia or dex treated A549 cells. Taken all together, these data indicated that dex and/or hypoxia significantly up-regulated the expression of stomatin *in vivo* and *in vitro*, which could stabilize membrane-associated actin in AEC. We suppose that the up-regulation of stomatin by hypoxia and dex may enhance the barrier function of alveolar epithelia and mediate the adaptive role of GC to hypoxia.

## Introduction

Hypoxia occurs in some environmental conditions such as ascent to a high altitude or more commonly in many pathologic conditions such as respiratory disorders and heart failure that can induce pulmonary oedema 1, 2. Hypoxic stress can affect several independent transcriptional regulations, in which hypoxia-inducible transcription factor-1 (HIF-1) is the most important transcription factor that primarily mediates the adaptive response to hypoxia by regulating the expression of its target genes 3. Release of glucocorticoid (GC) in response to hypoxic condition in animal and human has been extensively documented. GC plays an essential role in adaptation to hypoxic environments and homeostatic regulation. Synthetic GC, such as dexamethasone (dex), has been widely used to prevent and treat high mountain sickness (such as hypoxia-induced pulmonary oedema) 4, 5 and severe tissue damages caused by hypoxia and ischemia 6. It has been reported that GC alleviates pulmonary oedema and lung injury through multiple mechanisms, such as reducing the leaking of capillaries 2, inhibiting inflammatory response in lung, enhancing Na^+^ transport and alveolar fluid reabsorption in AEC 7– 9. The effects of GC are mediated by the GC receptor (GR), a transcription factor that belongs to the nuclear receptor superfamily. On binding GC, GR can modulate gene transcription through either a direct binding to GRE in the promoter region of target genes or interaction with other transcription factors, such as AP-1 and NF-κB 10, 11. In recent years, researchers pay more attention to the expression of genes and cellular responses induced by GC and hypoxia 12, 13. However, until now few genes were known to be regulated by both GC and hypoxia and involved in the GC action in tissue-specific and systemic adaptation to hypoxia.

Stomatin was first identified in human erythrocytes and absence of the protein is associated with a major abnormality in cation permeability in erythrocytes and a form of haemolytic anaemia known as overhydrated hereditary stomatocytosis (OHSt) 14, 15. Now it is known that stomatin is widely expressed in many cell types in mice and human beings. As an important lipid raft protein, stomatin is localized in sphingolipid/cholesterol-rich detergent-resistant membrane domains, and is involved in membrane organization and cholesterol-dependent regulatory processes 16–18. Stomatin also has been proposed to interact with several membrane proteins to regulate their biological activities although the precise mechanism is still unknown. For example, stomatin expressed in sensory neurons modulated the physiological properties of acid sensing ion channel (ASIC) and played a role in response to nociceptive, taste, and mechanical stimuli 19, 20. Stomatin was also found to co-localize with actin microfilaments in human amniotic epithelial cells (HAEC) and red blood cells 21, 22 and might function as a cytoskeletal anchor. Moreover, stomatin has been proved to bind to the glucose transporter GLUT-1 to decrease the rate of glucose uptake 23–25, and to be involved in storage-associated vesiculation in red blood cells 26. Although stomatin is an important functional protein, its regulation under physiological and pathological conditions is largely unknown. Our recent study found that hypoxia up-regulated the expression of stomatin in the brain 27. However, the effect of hypoxia on the expression of stomatin was not observed in endothelial cells 28. Therefore, it is interesting to ask whether the regulation of stomatin by hypoxia has cell type specificity. Recently, the expression of stomatin induced by dex was reported in HAEC 29 and A549 cells by microarray analysis 30. However, the mechanism and significance of up-regulation of stomatin remains to be determined. In this study, we investigated the effects of hypoxia and dex on the expression of stomatin in A549 cells, primary AEC and the lung of rats. It was found that hypoxia and dex alone or in combination up-regulated the expression of stomatin *in vitro* and *in vivo*. The mechanism of up-regulation of stomatin and the relationship between the expression of stomatin and membrane associated actin cytoskeleton in A549 cells were also investigated.

## Materials and methods

### Cell culture and hypoxia exposure

Human lung adenocarcinoma epithelial A549 cells were cultured routinely in DMEM/F-12 medium containing 10% newborn bovine serum (NBS, Gibco, Carlsbad, CA, USA) at 37°C in a 5% CO_2_ incubator. Rat AEC were isolated, purified and cultured as described previously 31. Briefly, the lungs of the anaesthetized Sprague-Dawley rats were lavaged, minced and treated enzymatically with 0.25% trypsin and 250 mg/ml deoxyribonuclease I. The obtained cell suspension was purified by discontinuous Percoll density gradient centrifugation. The cell viability estimated by trypan blue exclusion was more than 90%. The isolated alveolar type II cells were cultured in DMEM/F-12 medium supplemented with 10% fatal bovine serum (FBS) and 100 U/ml penicillin. Culture medium was changed 24 hrs after isolation and then on alternate days. Cells formed a confluent monolayer on the fourth day. Before experiments, the medium was replaced with DMEM/F-12 medium containing 10% dextran-coated charcoal treated NBS or FBS to avoid possible interference with serum steroids. Cells were then placed in an anaerobic system (Forma Scientific Inc, Marietta, OH, USA) containing 1% O_2_, 5% CO_2_, 94% N_2_ for hypoxia exposure with or without dex treatment 32.

### Animals and hypoxia exposure

The protocol for the study was approved by the Institutional Animal Care Committee of the Second Military Medical University in Shanghai, China. The animal care facility is accredited by the Association for Assessment and Accreditation of Laboratory Animal Care. Sprague-Dawley rats, weighing from 200 to 250 g, were obtained from Shanghai SLAC laboratory animal company. All animals were acclimatized in our animal laboratory for at least 7 days before the experiment, being fed with standard laboratory chow and water. The randomly selected rats were put in a normobaric hypoxia chamber (40 L, Yangyuan hyperbaric oxygen chamber company, Shanghai, China) and flushed with 8% O_2_ (a gas mixture of 8% O_2_ and 92% N_2_) for different times. After hypoxia exposure, animals were immediately anaesthetized and killed, and lungs were isolated for further experiments.

### Adrenalectomy and dex supplement

The animals were anaesthetized by intraperitoneal injection of 3% pentobarbital sodium (1 ml/kg BW), and adrenal glands were removed by the dorsal approach as described by Fleshner *et al*. 33. Sham animals underwent the same surgery except that the adrenal glands were left intact. Adrenalectomized (Adx) rats were given 0.9% saline ad libitum to compensate for sodium loss after the operation and allowed to recover for 1 week before dex supplement and/or hypoxia exposure.

The adx rats were injected intramuscularly with 5 mg/kg body weight of dex (Sigma-Aldrich Corp., St. Louis, MO, USA) dissolved in 0.9% NaCl solution. Control adx rats were treated with 0.9% NaCl alone 34. At the same time of dex supplement, hypoxia exposure was done as described above. Rats were anaesthetized and killed at 12 hrs after treatments and lungs were isolated for further experiments.

### RNA extraction and real-time PCR analysis

Total RNA was isolated using TRIzol reagent (Invitrogen, Carlsbad, CA, USA). Two micrograms of total RNA was reversely transcribed using reverse transcription reagents (Fermentas, Vilnius, Lithuania) in accordance with the manufacturer's instructions. Stomatin mRNA was analysed in triplicate using SYBR Green PCR Master Mix (Toyobo, Osaka, Japan) on Mastercycler ep realplex (Eppendorf, Hamburg, Germany). Primers used were: Stomatin (human) sense 5′-AGAGCTCCTGGTCCTCAA-3′, antisense 5′-TCTGTCCATCCAGCCAATG-3′. Stomatin (rat) sense 5′-CCCTGGCTGTGGCAAATA-3′, antisense 5′-GGGAAGACAATGGTGGAGT-3′. Amplification of stomatin cDNA was normalized to β-actin (internal control).

### Western blot analysis

The protein extracts from cells or tissues were electrophoresed on 10% SDS-polyacrylamide gel and transferred to nitrocellulose membrane. After being blocked with 5% nonfat milk in tris-buffered saline tween-20 (TBST), the membranes were immune-blotted with a mouse anti-stomatin antibody (1:1000, Santa Cruz, Dallas, TX, USA) or a mouse anti-β-actin monoclonal antibody (1:10000, Sigma-Aldrich Chemicals) overnight at 4°C followed by antimouse horseradish peroxidase conjugated secondary antibody (1:5000, Rockland Immunochemicals, Philadelphia, PA, USA). The blots were visualized by ECL reagent kit (Pierce, Rockford, IL, USA) following the manufacturer's recommended protocol and the relative expression of the protein bands of stomatin (31 kD) was quantified by scanning densitometry using β-actin as internal control.

### Construction of promoter-luciferase reporter plasmids, site-directed mutagenesis and reporter assay

For cloning of stomatin promoter-driven reporter plasmid, genomic DNA was acquired from A549 cells using MagExtractor-Genome kit (Toyobo) according to the manufacturer's instruction. DNA sequence was amplified *via* PCR using the following primers, sense 5′-AGTGGGTACCAGGACTGTTTGTGACAA-3′,antisense 5′-GAAGATCTTCAGGAAGCAGCGGTGTCTG-3′. PCR reactions were performed with PrimeSTAR® HS DNA polymerase (Takara, Dalian, China). The reaction condition was:one cycle 98°C for 1 min.; 30 cycles 98°C for 10 sec., 58°C for 15 sec., 72°C for 2 min. and 40 sec.; one cycle 72°C for 7 min. The amplification product was ligated to the blank luciferase reporter plasmid pGL3-Basic (Promega, Madison, WI, USA) *via Acc*65I and *Bgl*II restriction sites introduced by the primers. The constructed reporter plasmid containing about 2 kb (-1782 to +244) of promoter sequence of human stomatin gene was named STOM (-1782 to +244)-luc (STOM-1782) and the following truncated fragments containing the initial truncated position to +244 were named STOM-initial numbers separately, which were constructed using STOM-1782 as template and the forward primers were as follows:

STOM-1630: 5′-TAGGGTACCAACAAGCACTGGCCTCGAG-3′, STOM-1246: 5′-ACGGGTACCACACCACCCCACCTGG-3′, STOM-288: 5′-TATCGGTACCCTATCCTCACCATCTGCTC-3′, STOM-162: 5′-TACAGGTACCAGCCCACAGAACTAGCG-3′, STOM-10: 5′-ATGTGGTACCTGGGTCTTGTGCCTGTG-3′. The sequences of all constructed plasmids were confirmed by sequence analysis. Deletion mutants whose the putative GR binding sites were deleted were made with the PrimeSTAR® HS DNA polymerase (Takara). To remove the five conserved base pairs in the putative GRE sequences, the following primers were employed. mGRE1, sense: 5′-CACGCCCCTCAGCCCACTAGCGGGAAGTGACTGCGA-3′, antisense: 5′-TCGCAGTCACTTCCCGCTAGTGGGCTGAGGGGCGTG-3′. mGRE2, sense: 5′-GCAGTCGCGCCGTGGAGGGAGGGCGGGGATTGGG-3′, antisense: 5′-CCCAATCCCCGCCCTCCCTCCACGGCGCGACTGC-3′. mGRE3, sense: 5′-CGGCAATCTGGGTCTTTCTGGCTCCTCAGGGCA-3′, antisense: 5′-TGCCCTGAGGAGCCAGAAAGACCCAGATTGCCG-3′. mGRE4, sense: 5′-CAGCATGGCCGAGAAGCGGCACGGGACTCCGAAG-3′, antisense: 5′-CTTCGGAGTCCCGTGCCGCTTCTCGGCCATGCTG-3′. mNF-κB, sense: 5′-TGCCTCTGGCTCCTCAGGCCCGGCGGCTCCGGGTTT-3′, antisense: 5′-AAACCCGGAGCCGCCGGGCCTGAGGAGCCAGAGGCA-3′. mAP-1, sense: 5′-GTGAGTCCCGCGTCCCCCTCCCCCGTGGACCGAGCC-3′, antisense: 5′-GGCTCGGTCCACGGGGGAGGGGGACGCGGGACTCAC-3′.

Correct assembly of the deletion was confirmed by sequence analysis. For transient transfection reporter assay, A549 cells were seeded onto 48-well plates. Twenty-four hours later, cells were transiently transfected with the constructed promoter vectors (0.5 μg/well) using JetPEI (Polyplus-transfection, Illkirch, France) according to the manufacturer's instructions. Twenty-four hours later, cells were treated with dex for 24 hrs and lysed for detecting the reporter gene activity using dual luciferase assay kit (Promega).

### F-actin staining

F-actin filaments in A549 cells were stained with 3 μg/ml molecular probes phalloidin-tetramethylrhodamine isothiocyanate (Sigma-Aldrich), mounted with the anti-fade reagent and then examined by a laser-scanning confocal microscope (Leica, Wetzlar, Hesse, Germany).

### EGFP-tagged stomatin vector construction, transient transfection and RNA interference

The expression vector encoding for human stomatin (pEGFP-N3-stom) and blank vector (pEGFP-N3) were constructed as described by Umlauf *et al*. 18. EGFP-tagged stomatin plasmid (2 μg/well) was transiently transfected using JetPEI (Polyplus-transfection). Subsequent experiments were performed after 24 hrs of transfection.

siRNAs to stomatin and the scrambled siRNAs were designed and obtained from GenePharma (Shanghai, China). The sequences for stomatin-siRNA were 5′-GUGGCGUUCUCAUUCUUAUTT-3′(sense), and 5′-AUAAGAAUGAGAACGCCACTT-3′ (antisense) respectively. A549 cells were transfected with a final concentration of 1 or 3 nM of siRNA using INTERFERin™ (PolyPlus-Transfection) for 12 hrs according to the manufacturer's instruction. Then the medium was changed and A549 cells were allowed to recover for 12 hrs before subsequent dex treatment or hypoxia exposure.

### Statistical analysis

Data were presented as mean ± S.E. and analysed using one-way anova in the SPSS13.0 software package. Differences were considered statistically significant at *P* < 0.05.

## Results

### Hypoxia up-regulated stomatin expression

**Fig. 1:**
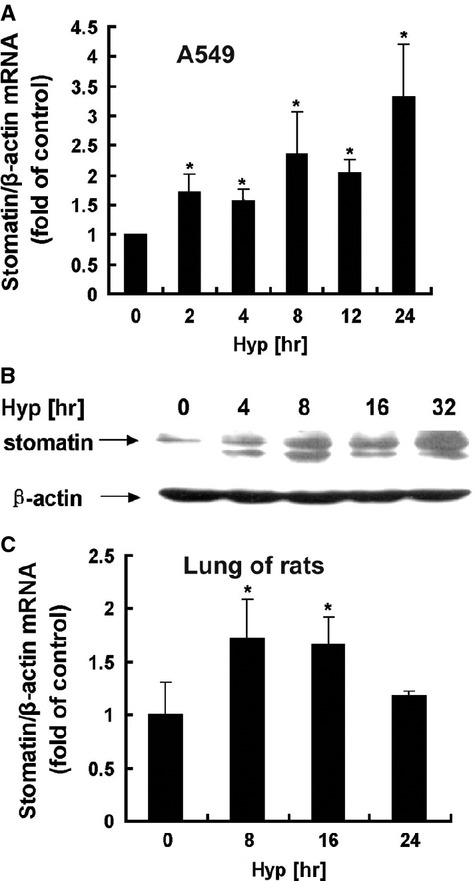
Hypoxia up-regulated stomatin expression. A549 cells were cultured in a normoxia incubator (with 5% CO_2_, 20% O_2_) or a hypoxia incubator (with 1% O_2_, 5% CO_2_, 94% N_2_) for different times. The levels of stomatin mRNA (**A**) and protein (**B**) were assessed by real-time PCR and Western blot. (**C**) Adult male Sprague-Dawley rats were put in a normal pressure hypoxia chamber filled with 8% O_2_ and 92% N_2_ for 8, 16 and 24 hrs, and rats in the control group stayed in normoxic environment (*n* = 9).The levels of stomatin mRNA were assessed by real-time PCR. β-actin was used as a normalization control for real-time PCR and Western blot. *P < 0.05 *versus* 0.

We first examined the expression of stomatin in A549 cells under normoxic or hypoxic (1% O_2_) conditions for different times by RT PCR and Western blot. We found that hypoxia significantly up-regulated stomatin at mRNA and protein levels in a time-dependent fashion (Fig. 1A and B). Increased stomatin mRNA in cells exposed to hypoxia was detected as early as 2 hrs and lasted for at least 24 hrs. Stomatin mRNA in cells exposed to hypoxia for 12 hrs was about twofold of that in the normoxic control (*P* < 0.05).

We further examined stomatin expression in the lung of rats after exposure to hypoxia (8% O_2_) for different times to determine if the effects of hypoxia on the expression of stomatin also occurred *in vivo*. As shown in Figure 1C, hypoxia also significantly up-regulated the expression of stomatin mRNA in the lung of rats (1.7-fold of the normoxic control after hypoxic exposure for 8 hrs, *P* < 0.05).

### Dex induced the expression of stomatin through GR in A549 cells

**Fig. 2:**
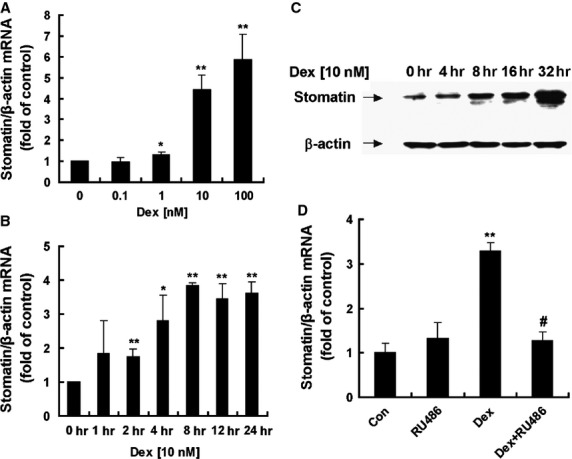
Dex induced the expression of stomatin through GR in A549 cells. The levels of stomatin mRNA in A549 cells treated with 0.1–100 nM dex for 12 hrs or treated with 10 nM dex for different times were assessed by real-time PCR (**A** and **B**). Stomatin proteins in A549 cells treated with 10 nM dex for different times were assessed by Western blot analysis (**C**). Stomatin mRNA was detected in A549 cells treated with 10 nM dex in the absence or presence of 100 nM RU486 (**D**). β-actin was used as a normalization control for real-time PCR and Western blot. Data were represented as mean ± S.D. of three independent experiments. **P < 0.01, *P < 0.05 *versus* 0 or control, #*P* < 0.01 *versus* Dex.

**Fig. 3:**
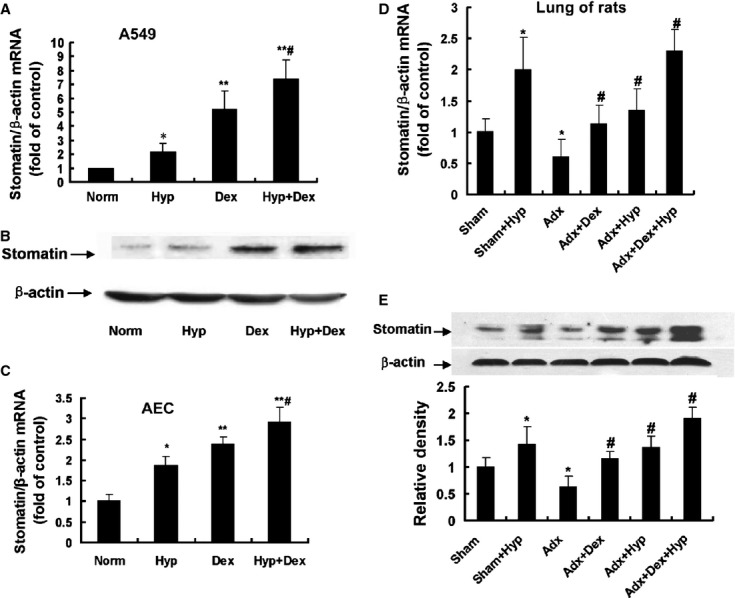
Hypoxia and dex had additive effects on up-regulation of stomatin expression *in vitro* and *in vivo*. A549 cells were exposed to hypoxia (1% O_2_, 5% CO_2_, 94% N_2_), or treated with 10 nM dex, or co-treated with hypoxia and dex for 16 hrs. Control cells were cultured in a normoxia incubator (with 5% CO_2_, 20% O_2_). The levels of stomatin mRNA and protein were assessed by real-time PCR (**A**) and Western blot (**B**). (**C**) Primary alveolar epithelial cells (AEC) were isolated and cultured to fourth or fifth passage. About 4 × 10^5^ cells/dish were treated as described above and stomatin mRNA were assessed. β-actin was used as a normalization control for real-time PCR and Western blot. Data were represented as mean ± S.D. of three independent experiments.*P < 0.05;**P < 0.01; #*P* < 0.01 *versus* Dex. Adult male rats with adx were exposed to hypoxia (8% O_2_ and 92% N_2_), or treated with dex (5 mg/kg), or co-treated with both hypoxia and dex for 12 hrs. Sham rats were treated with or without hypoxia (*n* = 6–9). The levels of stomatin mRNA and protein were assessed by real-time PCR (**D**) and Western blot (**E**). Stomatin level was normalized to that of β-actin. Values were expressed as mean ± S.D. *P < 0.05 *versus* sham, #*P* < 0.05 *versus* adx.

The effect of dex on stomatin expression in A549 cells was investigated using RT PCR and Western blot. Cells were treated with different concentrations of dex (0.1–100 nM) for 12 hrs, or 10 nM dex for different times. As shown in Figure 2A and B, dex increased the mRNA level of stomatin in a concentration- and time-dependent manner. The level of stomatin mRNA in A549 cells treated with 10 nM dex for 8 hrs was about 3.8-fold of that in control cells (*P* < 0.01). Ten nanomole dex also significantly increased stomatin expression at protein level in a time-dependent fashion (Fig. 2C). The stomatin mRNA induced by dex was significantly blocked by RU486, an antagonist of GR (Fig. 3D), indicating that the effects of dex were mediated through GR.

### Hypoxia and dex had additive effects on up-regulation of stomatin expression *in vitro* and *in vivo*

To study the combined effects of hypoxia and dex on stomatin expression, A549 cells and primary AEC were used. As shown in Figure 3A, treatment of A549 cells with hypoxia or dex individually increased stomatin mRNA to 2.1- and 5.2-fold of those in control cells. Treatment of cells with hypoxia and dex led to a further increase in stomatin mRNA (7.3-fold of that in control cells, *P* < 0.01). Similar results were observed at protein levels in A549 cells (Fig. 3B) and mRNA levels in AEC (Fig. 3C).

It has been reported that *in vivo* hypoxia results in releasing high levels of endogenous GC. In this study we used adx rats in which endogenous adrenal hormones (mainly GC) were removed to examine the effects of hypoxia on the expression of stomatin with or without supplying with dex (5 mg/kg). As shown in Figure 3D, stomatin mRNA in adx group was decreased to about 50% of that in sham group in normoxia, indicating that adrenal cortical hormones are essential for maintaining the normal level of stomatin. Hypoxia significantly stimulated the expression of stomatin both in sham group (twofold of that in the sham group, *P*<0.05) and in adx group (2.2-fold of that in the adx group, *P* < 0.05). Moreover, administration of adx rats with dex for 12 hrs significantly increased the expression of stomatin mRNA (about 1.8-fold of that in the adx group) in normoxia, and further increased stomatin expression (about 3.7-fold of that in the adx group) in hypoxia. Similar results were observed at protein levels in different groups (Fig. 3E). These results indicated that hypoxia and dex had additive effect on the up-regulation of stomatin expression *in vitro* and *in vivo*.

### Dex, but not hypoxia, increased the activity of stomatin promoter reporter gene [STOM(-1782 to +244)-luc] in A549 cells

**Fig. 4:**
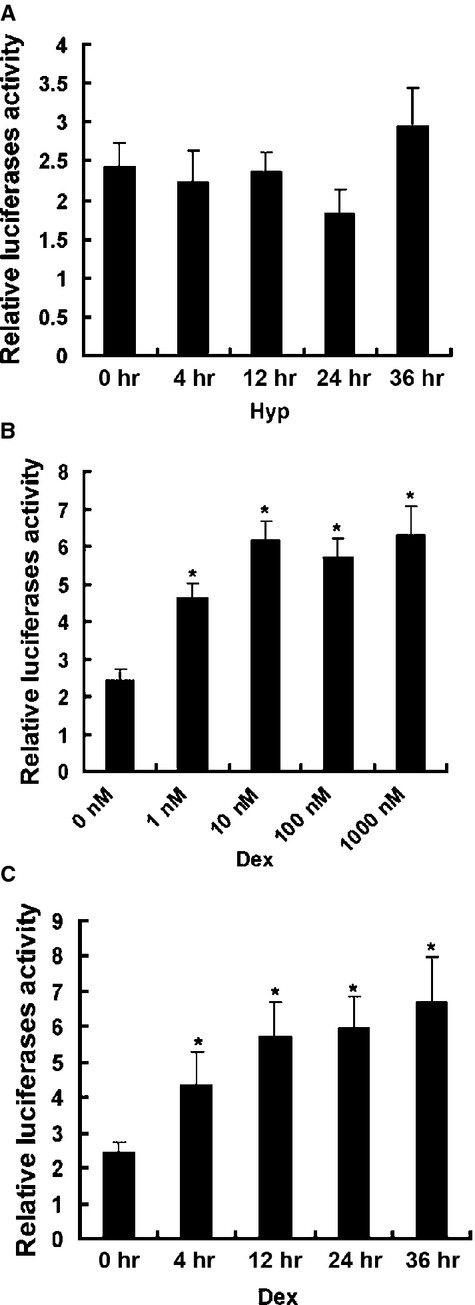
Dex induced stomatin promoter activity. A549 cells were tansfected with stomatin promoter reporter gene [STOM(-1782 to +244)-luc] for 24 hrs. The promoter activities of stomatin in cells were determined by luciferase analyses after cells were exposed to hypoxia for different times (**A**) or treated with 1–1000 nM dex for 24 hrs (**B**) or with 100 nM dex for different times (**C**). Firefly luciferase activities were normalized to renilla activities. All data were expressed as mean ± S.D. of three experiments, each in triplicate. *P < 0.05 *versus* control.

To determine whether hypoxia or dex regulates the stomatin gene at transcriptional level, a stomatin reporter plasmid STOM (-1782 to +244)-luc containing about 2 kb (-1782 to +244) of promoter sequence of human stomatin gene was constructed and transfected into A549 cells. The reporter activity was determined by luciferase assay after cells were exposed to hypoxia or treated with dex for different times. The results showed that reporter activity did not change obviously after cells were exposed to hypoxia (Fig. 4A). However, dex induced the luciferase activity in a concentration- and time-dependent manner (Fig. 4B and C). Significant increase of reporter activity in cells treated with 100 nM dex was observed at 4 hrs with a maximal level at 36 hrs of about 2.8-fold of that in control cells (*P* < 0.05), indicating that dex can up-regulate stomatin expression at transcriptional level.

### A potential GRE mainly contributed to the response of stomatin promoter reporter gene to dex

**Fig. 5:**
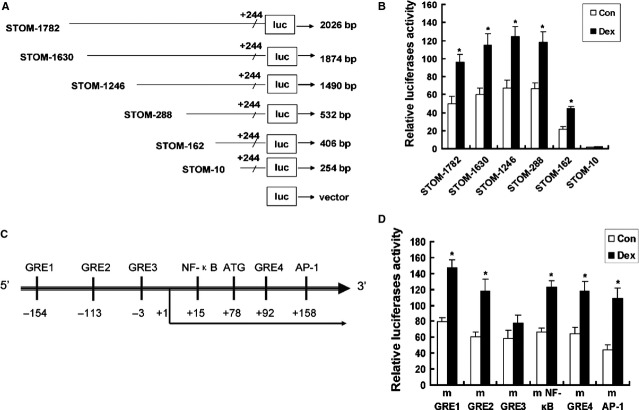
GRE3 contributed to the response of stomatin promoter reporter gene to dex. (**A**) A series of 5′ deletions of the stomatin promoter were cloned into pGL3-Basic reporter plasmid. (**B**) A549 cells were transiently transfected with the stomatin promoter deletion constructs for 24 hrs. Cells were harvested for luciferase assay after being treated with vehicle or 100 nM dex for 24 hrs. (**C**) Several candidate transcription factor binding sites were selected, and corresponding deletion variants were constructed. Wild-type STOM-288 was used as template to make variant promoter vectors. (**D**) The variant promoter vectors were transfected into cells and the luciferase activities in cells were determined as above. Values were represented as mean ± S.D. of three representative experiments. *P < 0.05 *versus* Con.

To determine the response elements which are responsible for dex induction, a series of 5′ deletion mutants of the stomatin promoter were constructed (Fig. 5A) and linked to luciferase. A549 cells were transfected with these mutated stomatin promoter-luc vectors for 24 hrs, and treated with vehicle or 100 nM dex for additional 24 hrs. The activities of luciferase were determined by luciferase assay. As shown in Figure 5B, 5′ deletion of the promoter to −162 markedly decreased the basal promoter activity, but had no effect on dex inducibility. However, further deletion from −162 to −10 markedly decreased both the basal and dex induced promoter activity, suggesting that this region (between −162 and −10) was not only a core promoter region to sustain the basal transcriptional activities, but also essential to the induction of stomatin by dex. We did not examine the induction effect of dex between −10 and +244 because the basal promoter activity in this region was too low to be detected. The possibility that the region −10 to +244 contains the potential GREs cannot be excluded.

There were four potential GREs, one AP-1 and one NF-κB binding site within the −162 to +244 region of stomatin gene by transcription element search software analysis (Fig. 5C). Using STOM-288 as template, we constructed the corresponding mutant reporters containing these potential transcription factor binding sites and transfected them into A549 cells separately. As shown in Figure 5D, only GRE3 mutant showed a significant decrease in the induction of reporter activity in response to dex treatment. All other mutants showed an intact basal activity and comparable dex induction. These results indicated that the GRE3 binding site mainly contributed to the responsiveness of stomatin promoter to dex.

### Up-regulation of stomatin by hypoxia and dex stabilized membrane-associated actin cytoskeleton in A549 cells

**Fig. 6:**
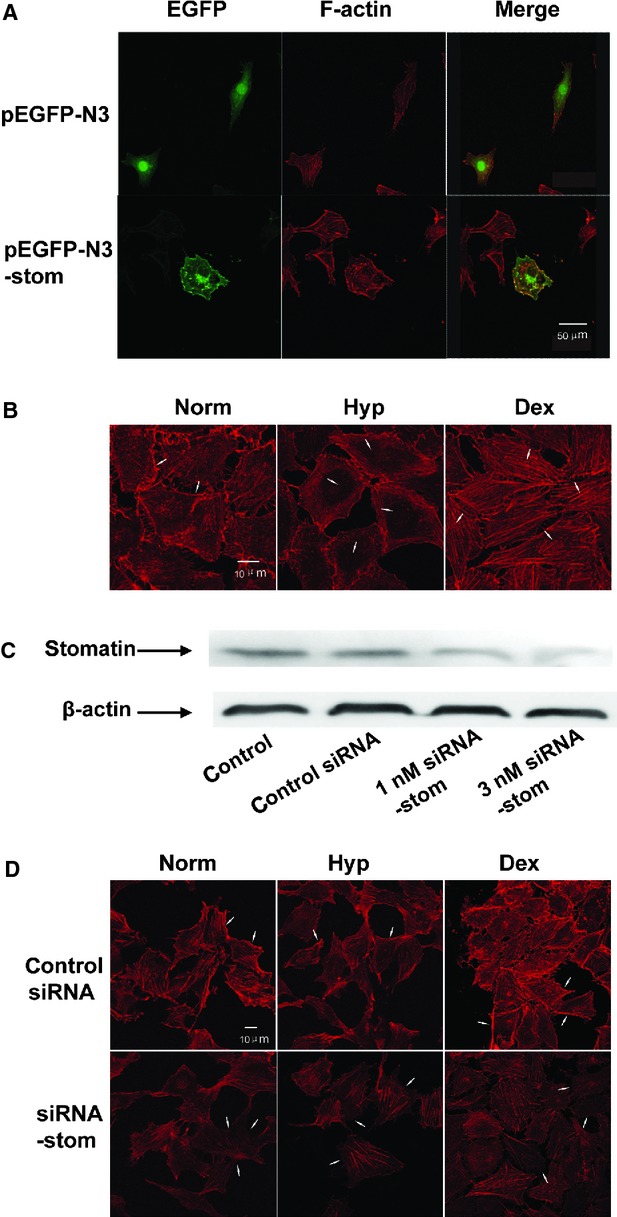
Stomatin stabilized membrane-associated actin. (**A**) A549 cells were transiently transfected with blank vectors pEGFP-N3 or pEGFP-N3-stom. Images of stomatin tagged by EGFP (green) and actin filaments labelled by rhodamine-phalloidine (red) were visualized and captured using confocal microscopy. (**B**) A549 cells were cultured in normoxia or exposed to hypoxia or treated with 100 nM dex for 12 hrs. Actin filaments were viewed using confocal microscopy (100×). (**C**) Stomatin protein in A549 cells (control) or cells transfected with negative control siRNAs (control siRNA) or with 1 or 3 nM stomatin siRNAs (siRNA-stom) were analysed by Western blot. β-actin was detected as an internal control. (**D**) A549 cells transfected with 3 nM siRNA-stom or control siRNA were cultured either in normoxic or hypoxic environment or treated with 100 nM dex. Actin filaments were viewed using confocal microscopy (60×).

As actin cytoskeleton plays an important role in alveolar epithelial barrier and in many cellular functions, we examined the relationship between stomatin and actin cytoskeleton. We first studied the cellular localization of actin and stomatin in A549 cells transiently transfected with blank vector (pEGFP-N3) or EGFP-tagged stomatin expression vector (pEGFP-N3-stom). The results showed that EGFP-tagged stomatin protein was clustered in the membrane and around the perinucleus. Stomatin proteins in the membrane were co-localized with membrane-associated actin filaments labelled by rhodamine-phalloidine in A549 cells (Fig. 6A).

Then we investigated the effect of hypoxia or dex treatment on actin cytoskeleton for 12 hrs in A549 cells. As shown in Figure 6B, in control cells, it formed a peripheral actin ring (shown by arrows) and homogeneous distribution of actin in the cytoplasm of cells in normoxia. Hypoxia exposure caused a decrease in actin in cytoplasm, but did not significantly affect membrane-associated actin filaments. Dex led to reorganization of actin cytoskeleton with actin polymerization and formation of stress fibre (shown by arrows).

We further inhibited the expression of stomatin in A549 cells with 1 or 3 nM stomatin small interfere RNA (siRNA-stom; Fig. 6C), and investigated the relationship of the up-regulated stomatin expression by hypoxia or dex and membrane-associated actin cytoskeleton in A549 cells. As shown in Figure 6D, transfection itself resulted in formation of a few stress fibres in the transfected cells (control siRNA and siRNA-stom), knockdown of stomatin expression by siRNA-stom decreased the dense of peripheral actin ring in normoxic cells, hypoxic cells or dex-treated cells as compared with that in cells transfected with control siRNA (shown by arrows). These observations indicated that up-regulation of stomatin by hypoxia and dex could stabilize membrane-associated actin and increase epithelial cell integrity.

## Discussion

In this study we found for the first time that hypoxia could significantly up-regulate stomatin expression in A549 cells, primary AEC, and the lung of rats. Transient transfection reporter assays showed that hypoxia had little effect on stomatin promoter activities in A549 cells, in line with the fact that there is no classical HIF-responsive element in the promoter region of the stomatin gene analysed by transcription factor binding sites prediction software. Further experiments are needed to clarify whether other regions beyond this cloned promoter region may contribute to the hypoxia regulation of stomatin transcription or the up-regulation of stomatin by hypoxia occurs at post-transcriptional level, such as increasing stability of stomatin mRNA.

In addition to hypoxia, we observed that dex also induced the expression of stomatin *in vivo* and *in vitro*. This effect was mediated by the GR. Moreover, dex induced the luciferase activity of stomatin promoter reporter gene. There are four potential GREs in the promoter region of the stomatin gene and mutational studies showed that among these four potential GREs, only GRE3, an imperfect consensus sequence (CTTGTGCCTCTGG), was important for the effect of dex. As dex could result in a four- to fivefold increase in the levels of stomatin mRNA and the maximal induction of the activity of stomatin promoter reporter gene was only 2.8-fold, other GREs beyond this cloned promoter region may also contribute to the GR transcriptional regulation of stomatin 35, 36. Moreover, there is a possibility that dex also increases the expression of stomatin at post-transcriptional level.

Recent studies have found that there existed membrane raft actin deficiency and altered Ca^2+^-induced vesiculation in stomatin-deficient OHSt patients, and when stomatin was expressed in Madin-Darby canine kidney cells, actin association with the membrane was increased 22. These findings imply that the stomatin-actin association is important in maintaining the structure and function of membrane rafts in red cells. In this study we found that stomatin protein was co-localized with membrane-associated actin filaments in A549 cells, and exposure of A549 cells to hypoxia caused a decrease of actin in cytoplasm, but did not significantly affect membrane-associated actin filaments. The changes of actin were not due to a decrease of actin expression in hypoxia-exposed cells as levels of actin protein in normoxic and hypoxic cells determined by Western blot were comparable (data not shown). Similar results were also reported by other groups that hypoxia induced a disorganization of actin with a loss of stress fibres, but did not affect cortical and apical actin in AEC 37.Our study demonstrated that inhibition of stomatin expression by stomatin siRNA decreased the dense of peripheral actin ring in control cells, hypoxic cells and dex-treated cells. These results indicate that stomatin play a role in stabilizing membrane-associated actin. It is known that membrane-associated actin cytoskeleton is involved in many cellular functions, such as the maintenance of cell shape, polarity, flexibility, cellular integrity, location of membrane proteins, linkage and organization of rafts in the cell membrane 38, 39. Actin in alveolar epithelium also plays an extensive regulatory role by interacting directly or indirectly with Na^+^, K^+^-ATPase, Na^+^ transporters, tight junction proteins and cell-cell adhesion molecules 40, 41. Therefore, up-regulation of stomatin by hypoxia and dex might enhance the barrier function of alveolar epithelia by stabilizing membrane-associated actin.

As a raft protein, stomatin is believed to combine several other membrane proteins besides actin, such as ASIC in sensory neuron 20, glucose transporter GLUT-1^23^ and so on, to regulate their biological activities although the precise mechanism is still unknown. Therefore, it will be interesting to determine if hypoxia and dex can affect the activities of these proteins by up-regulating the expression of stomatin thereby influencing their physiological and pathological significance.

In summary, this study showed that hypoxia and dex significantly up-regulated the expression of stomatin individually or in combination *in vitro* and *in vivo*. The effects of dex occurred at transcriptional level. Up-regulated expression of stomatin by hypoxia and dex could stabilize membrane-associated actin cytoskeleton in A549 cells, which might enhance the barrier function of alveolar epithelia and mediate the adaptive role of GC to hypoxia.
